# Compensation versus deterioration across functional networks in amnestic mild cognitive impairment subtypes

**DOI:** 10.1007/s11357-024-01369-9

**Published:** 2024-10-05

**Authors:** Benxamín Varela-López, Montserrat Zurrón, Mónica Lindín, Fernando Díaz, Santiago Galdo-Alvarez

**Affiliations:** 1 Department of Clinical Psychology and Psychobiology, Universidade de Santiago de Compostela (USC), Santiago de Compostela, Spain; 2 Cognitive Neuroscience Research Group (Neucoga-Aging), Instituto de Psicoloxía, USC (IPsiUS), Health Research Institute of Santiago de Compostela (IDIS), Santiago de Compostela, Spain

**Keywords:** Resting-state functional magnetic resonance imaging, Amnestic MCI, Amnestic MCI subtypes, Default mode network, Fronto-parietal control network

## Abstract

Functional connectivity studies to detect neurophysiological correlates of amnestic mild cognitive impairment (aMCI), a prodromal stage of Alzheimer’s disease, have generated contradictory results in terms of compensation and deterioration, as most of the studies did not distinguish between the different aMCI subtypes: single-domain aMCI (sd-aMCI) and multiple-domain aMCI (md-aMCI). The present study aimed to characterize the neurophysiological correlates of aMCI subtypes by using resting-state functional magnetic resonance imaging. The study included sd-aMCI (*n* = 29), md-aMCI (*n* = 26), and control (*n* = 30) participants. The data were subjected to independent component analysis (ICA) to explore the default mode network (DMN) and the fronto-parietal control network (FPCN). Additionally, seed-based and moderation analyses were conducted to investigate the connectivity of the medial temporal lobe and functional networks. aMCI subtypes presented differences in functional connectivity relative to the control group: sd-aMCI participants displayed increased FPCN connectivity and reduced connectivity between the posterior parahippocampal gyrus (PHG) and medial structures; md-aMCI participants exhibited lower FPCN connectivity, higher anterior PHG connectivity with frontal structures and lower posterior PHG connectivity with central-parietal and temporo-occipital areas. Additionally, md-aMCI participants showed higher posterior PHG connectivity with structures of the DMN than both control and sd-aMCI participants, potentially indicating more severe cognitive deficits. The results showed gradual and qualitative neurofunctional differences between the aMCI subgroups, suggesting the existence of compensatory (sd-aMCI) and deterioration (md-aMCI) mechanisms in functional networks, mainly originated in the DMN. The findings support consideration of the subgroups as different stages of MCI within the Alzheimer disease continuum.

**Supplementary Information:**

The online version contains supplementary material available at 10.1007/s11357-024-01369-9.

## Introduction

The onset of Alzheimer’s disease (AD), the most frequent cause of dementia, may occur at least 20–30 years before the appearance of the first clinical symptoms [1]. Research efforts have therefore focused on early diagnosis to provide the opportunity for the best therapeutic approaches to be applied. In this respect, mild cognitive impairment (MCI) has been defined as a prodromal stage characterized by objective cognitive impairment but with the ability to carry out daily life activities still intact [2–4].

Due to the heterogeneous characteristics of MCI, classification of different subtypes has been proposed [2, 4]. Patients are classified regarding which cognitive domain is impaired (memory or others such as executive functions, language, visuospatial skills, …) and the number of domains affected (single domain or multidomain). Thus, a patient could be classified into one of the four possible clinical subtypes: (i) single-domain amnestic MCI, (ii) multi-domain amnestic MCI, (iii) single-domain non-amnestic MCI, or (iv) multi-domain non-amnestic MCI [4].

Specifically, amnestic MCI (aMCI) may correspond to the prodromal stage of AD, with an annual conversion rate to dementia of about 10–15% [4, 5]. Furthermore, the two subtypes of aMCI show different rates of conversion to AD dementia [6]. In a 4-year follow-up study, the rate of conversion to multidomain aMCI (md-aMCI) was 77%, while for single-domain aMCI (sd-aMCI) the rate was 24% [7]. These data support the consideration of sd-aMCI and md-aMCI as different transitional, prodromal stages in the AD continuum [4, 6].

Resting-state functional magnetic resonance imaging (rs-fMRI) has been proposed as a promising non-invasive tool for characterizing neurocognitive indicators of the prodromal stages of AD [8, 9]. Indeed, the functional connectivity of large-scale brain networks, particularly the fronto-parietal control network (FPCN) and the default mode network (DMN), have been consistently found to be altered in aMCI and AD [10–12].

The FPCN comprises regions that are primarily involved in maintaining cognitive control, such as the anterior prefrontal cortex, anterior insular cortex, and anterior cingulate cortex. It also encompasses regions primarily associated with feedback in the context of cognitive control, including the dorsolateral prefrontal cortex, inferior parietal lobe, caudate nucleus, and lateral cerebellum [13]. Through its interactions with other brain networks, the FPCN enables execution of novel tasks in a rapid, adaptable manner [13].

The DMN comprises a set of brain regions characterized by robust functional connectivity at rest, including the medial prefrontal cortex (mPFC), posterior cingulate cortex (PCC), angular gyrus, and the precuneus [14]. Notably, at the start of task execution, the DMN is deactivated [14, 15]. Studies involving the DMN have revealed that the parahippocampal gyrus (PHG) serves as the primary hub within the medial temporal lobe (MTL) [16, 17]. In fact, it has been suggested that resting-state connectivity between the hippocampus and the posterior cingulate cortex is indirect and mediated by the PHG [17]. The PHG can be sub-divided into the posterior PHG (pPHG) (the parahippocampal cortex itself) and the anterior PHG (aPHG) (perirhinal/entorhinal cortices) [16, 18]. Multiple theoretical models suggest that the PHG plays a critical role in episodic memory, the main cognitive process affected in aMCI and AD dementia. The pPHG is strongly associated with recollection processes, specifically encoding and retrieving contextual information. On the other hand, aPHG activity is primarily involved in familiarity processes by encoding and retrieving specific item information [18]. These findings support the hypothesis that the MTL memory system represents a functional DMN subnetwork that is related to the cortical nodes of the DMN through PHG functional connections [16, 17]. Furthermore, alterations in the PHG may occur earlier along the AD continuum than alterations in the hippocampus [19, 20].

Studies comparing aMCI and control participants have shown both increases and decreases in DMN and FPCN functional connectivity. The reduced connectivity in both networks in aMCI participants relative to controls has consistently been interpreted as indicating impairment [11, 21]. Increased connectivity of FPCN has been interpreted as a compensatory phenomenon [21, 22]; however, there is no agreement regarding the interpretation of enhanced connectivity of the DMN, which has been associated with a compensatory process[22–25] and, by contrast, with an aberrant overexcitation pattern (i.e., a sign of impairment) [16, 26, 27].

Although several factors may contribute to the discrepancies between the findings of different studies, most rs-fMRI studies do not consider aMCI subtypes [28], even though these might correspond to two different stages of the AD continuum [29]. In fact, most studies generally reinforce the notion of different alterations in functional connectivity between aMCI subtypes, although with inconsistent results. Some researchers have reported that sd-aMCI participants exhibit higher connectivity of FPCN structures than md-aMCI participants and a gradual reduction in DMN connectivity between subtypes relative to healthy controls [30], whereas others have reported alteration of DMN connectivity only in md-aMCI, with no differences between sd-MCI and healthy controls [31]. Thus, further research is needed to assess the changes in the functional connectivity associated with each aMCI subtype, to better characterize these prodromal stages of AD, and to try to resolve the discrepancies in the current literature [32].

The present study aimed to compare the functional connectivity of the FPCN and the DMN in different aMCI subtypes (sd-aMCI and md-aMCI) and a control group, by using the rs-fMRI technique. For this purpose, independent component analysis (ICA) was conducted to estimate and compare the connectivity of FPCN and DMN at rest. Resting-state seed-to-voxel analyses of the PHG connectivity was also performed, considering the anterior and posterior functional subdivisions, to examine the role of the PHG in the communication between the MTL memory system and other networks and its functional differentiation. Additionally, post hoc causal analyses were conducted using neuropsychological scores associated with the affected regions/networks and drawing on prior knowledge of their functional role, to explore the potential links between the observed group differences in connectivity.

Considering the findings of previous studies that distinguished between subtypes [30, 31], we hypothesized gradual changes in connectivity across different subtypes of aMCI, anticipating greater alterations in both DMN (including PHG connectivity) and FPCN within the md-aMCI group. Moreover, we expected to observe compensatory effects that enhance network efficiency within the FPCN, while simultaneously detecting patterns of deterioration in the DMN across the aMCI continuum.

## Methods

### Sample

In this study, we examined a sample of 85 participants recruited from a larger group partaking in the longitudinal Compostela Aging Study (CompAS). The participants were referred to the CompAS from Primary Care Health Centres in Santiago de Compostela, Galicia, Spain [33]. The sample comprised 30 cognitively unimpaired (CU) participants, 29 participants diagnosed with sd-aMCI, and 26 participants diagnosed with md-aMCI.

The three groups were matched for age and sex but not years of formal education (*p* = 0.002). Demographic, behavioral, MRI volumetric, and neuropsychological scores for each group are summarized in Table 1.

**Table 1:** Mean values and standard deviations (SD, in brackets) of demographic, neuropsychological measures, and PHG gray matter volume

	Control group *N* = 30	sd-aMCI *N* = 29	md-aMCI *N* = 26	p*	Post hoc comparison
Age	66.17 (9.68)	66.51 (9.33)	68.92 (8.05)	0.480	NS
Years of education	12.83 (6)	11.14 (5.89)	7.85 (2.91)	0.002	0.001^b^/0.02^c^
Gender (female/male)	13/17	12/17	15/11	0.423	NS
General cognitive functioning					
MMSE	28.5 (1.81)	27.59 (2.35)	26.00 (2.67)	< 0.001	< 0.001^b^/0.012^c^
CAMCOG-R (total)	92.83 (8.65)	87.56 (10.24)	79.54 (7.84)	< 0.001	< 0.001^b^/ < 0.001^c^
Attention					
TMT-A (seconds)	52.23 (33.25)	56.21 (30.20)	84.35 (40.98)	0.002	0.001^b^/0.004^c^
CAMCOG-R (attention and calculation)	7.83 (1.56)	7.93 (1.31)	6.35 (1.87)	< 0.001	0.001^b^/ < 0.001^c^
Executive function					
TMT-B (seconds)	134.63 (88.24)	150.44 (105.55)	284.92 (129.34)	< 0.001	< 0.001^b^/ < 0.001^c^
CAMCOG-R (phonological verbal fluency)	16.13 (5.64)	13.17 (4.76)	8.85 (4.1)	< 0.001	< 0.001^b^/0.023^a^/0.002^c^
CAMCOG-R (executive function)	23.70 (14.69)	19.28 (4.85)	14.35 (3.43)	0.002	< 0.001^b^
Memory					
CVLT (immediate free recall)	51.07 (7.90)	36.45 (8.07)	33.5 (13.19)	< 0.001	< 0.001^ab^
CVLT (short-delay free recall)	11.00 (2.36)	5.48 (2.37)	5.69 (3.06)	< 0.001	< 0.001^ab^
CVLT (long-delay free recall)	11.57 (2.25)	5.58 (3.04)	5.00 (3.16)	< 0.001	< 0.001^ab^
CAMCOG-R (memory)	22.33 (2.70)	19.59 (4.20)	17.46 (4.10)	< 0.001	< 0.001^b^/0.006^a^/0.036^c^
Language					
BNT	50.87 (7.71)	46.80 (7.94)	38.65 (7.48)	< 0.001	< 0.001^bc^/0.046^a^
CAMCOG-R (semantic verbal fluency)	19.40 (6.38)	17.55 (3.01)	12.23 (2.76)	< 0.001	< 0.001^bc^
CAMCOG-R (language)	26.80 (2.55)	26.82 (1.47)	24.35 (1.96)	< 0.001	< 0.001^bc^
Depression					
Geriatric depression scale-15	2.30 (2.35)	2.07 (1.93)	2.62 (1.70)	0.608	NS
Functionality					
Lawton and Brody scale	7.37 (1.10)	6.65 (1.61)	7,11 (1.37)	0.138	NS
PHG GMv					
Left aPHG (mm^3^)	3539.38 (252.44)	3496.84 (299.86)	3353.35 (332.58)	0.056	NS
Left pPHG (mm^3^)	2177.31 (141.08)	2167.35 (150.89)	1999.85 (191.69)	< 0.001	< 0.001^bc^
Right aPHG (mm^3^)	3902.98 (263.29)	3885.37 (296.90)	3743.03 (232.26)	0.059	NS
Right pPHG (mm^3^)	1710.89 (149.67)	1715,16 (164.20)	1603.37 (173.26)	0.020	0.015^b^/0.013^c^

*sd-aMCI* Single-domain amnestic mild cognitive impairment, *md-aMCI* multiple-domain amnestic mild cognitive impairment, *PHG GMv* parahippocampal gyrus gray matter volume, *aPHG* anterior parahippocampal gyrus, *pPHG* posterior parahippocampal gyrus. Post hoc comparisons: ^a^Control group vs. sd-aMCI; ^b^Control group vs. md-aMCI group; ^c^sd-aMCI group vs. md-aMCI group; *ANOVA *p* value; *MMSE* Mini-Mental State Examination, *CVLT* California Verbal Learning Test, *CAMCOG-R* Cambridge Cognitive Examination, *BNT* Boston Naming Test, *TMT* Trail Making Test

This study was conducted according to the ethical standards outlined in the Declaration of Helsinki. All participants provided written informed consent prior to taking part in the study. To ensure the homogeneity of the sample, participants were excluded from the study if they had a prior diagnosis of depression or other psychiatric disturbances according to the Diagnostic and Statistical Manual of Mental Disorders, Fifth Edition criteria (DSM-5) [34]. Participants previously diagnosed with neurological disorders, including probable AD or other types of dementia, according to the National Institute of Neurological and Communicative Disorders and Stroke and the AD and Related Disorders Association (NINCDS-ADRDA) and DSM-5 criteria, were also excluded. Finally, individuals with a history of brain damage or brain surgery, previous chemotherapy, sensory or motor disturbances, or who had consumed substances that could affect the task performance were also excluded from the study.

Clinical, neurological, and neuropsychological examinations were conducted by general practitioners, cognitive neurologists, and neuropsychologists specialized in aging and dementia. MCI was diagnosed according to Petersen’s criteria, which include evidence of concern corroborated by an informant, poorer performance in one or more cognitive domains than expected for age and educational background, intact ability to carry out functional abilities, and non-fulfillment of diagnostic criteria for dementia according to NINCDS-ADRDA and DSM-5 criteria.

The cognitive assessment was conducted following the procedures outlined in [33]. Impairment in one domain was indicated when performance in two different tests for that domain was within the 1–2 standard deviation range below appropriate norms [35].

### Acquisition parameters and data pre-processing

Magnetic resonance imaging (MRI) was conducted using a Philips 3 T Achieva scanner (Philips Medical System). Structural imaging involved a sagittal T1-weighted 3D Magnetization Prepared Rapid Acquisition Gradient Echo (MPRAGE) sequence, while functional magnetic resonance images were acquired using a gradient echo-planar imaging (EPI) sequence sensitive to blood oxygen level–dependent (BOLD) contrast during the eyes-open resting state (while a fixation cross was presented following previous recommendations; [36]. Head movements were minimized with a vacuum cushion, and four dummy scans were discarded to establish steady-state magnetization. The MPRAGE sequence had a repetition time/echo time of 7.45 ms/3.40 ms, flip angle of 8°, voxel size of 1 × 1 × 1 mm, field of view of 240 × 240 mm^2^ and matrix size of 240 × 240 mm. The EPI sequence had a repetition time/echo time of 2000 ms/30 ms, flip angle of 87°, 37 interleaved slices, voxel size of 3 × 3 × 3.5 mm, field of view of 240 × 240 mm^2^, and matrix size of 80 × 80 mm.

A detailed description of the methodology used to preprocess functional and structural magnetic resonance images is provided in Online Resources 1.

### Independent component analysis

ICA was performed with the group ICA of fMRI toolbox (GroupICATv4.0b), the CONN 19c, ICN_Atlas [37] and the SPM12 toolboxes implemented in Matlab R2019a.

First, GroupICATv4.0b was used to estimate the number of independent components within the sample, as the CONN toolbox lacks this function. The minimum description length (MDL) criterion was used to reduce the data dimensionality and estimate the number of components, enhancing the efficiency of ICA [38]. The MDL criterion indicated 34 independent components in the sample (*n* = 85).

The first-level ICA was then conducted with the CONN toolbox and the 34 components determined by the MDL criterion. The following steps, derived from Calhoun’s group-level ICA approach [39] and implemented in CONN, were performed: variance normalization pre-conditioning, subject-level dimensionality reduction (64, default value in CONN), concatenation of the BOLD signal data along the temporal dimension for each subject/condition, group-level dimensionality reduction (targeting the 34 components estimated by the MDL criterion), fastICA1 [40], for independent spatial component estimation, and GICA3 back-projection, for individual subject-level spatial map estimation (refer to [39]; the official website of CONN and [41]for detailed information).

**Fig. 1:**
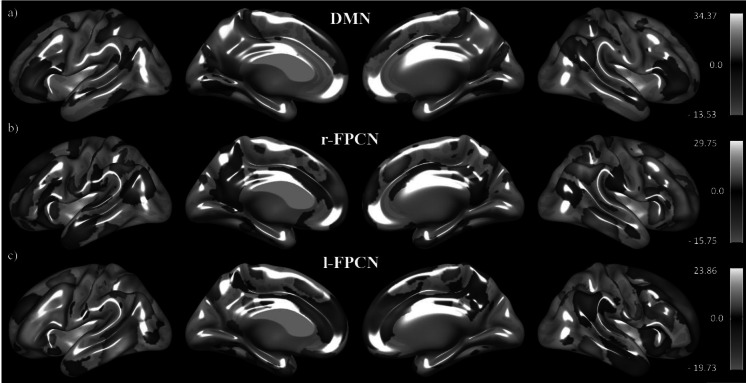
One-sample of the three independent components derived from the whole sample. DMN (**a**), right FPCN (**b**), and left FPCN (**c**). Results are significant at *p* < 0.05 FWE and FDR cluster-corrected in a combination with a threshold of *p* < 0.001 at the uncorrected voxel level. The colored bars represent *t* values derived from the one-sample analyses of connectivity maps, serving as a legend for the connectivity maps shown on the semi-inflated white matter surfaces

Three components of interest were identified according to a combination of visual and mathematical criteria by using the spatial involvement measure implemented in the ICN_Atlas toolbox [37] and the network atlas proposed by [42] as a template. The independent components corresponding to the DMN, left FPCN, and right FPCN of each participant were used for the second-level analyses (see Fig. 1).

In MCI or early-stage AD, atrophy tends to be distributed across various brain regions [43, 44]. Therefore, considering the diffuse nature of atrophy at early stages, and the fact that ICA is a whole-brain analysis, we concluded that it was not necessary to apply correction for atrophy in the ICA conducted in this study.

The significance level was set at *p* < 0.05 for the cluster level, in conjunction with an uncorrected voxel-level threshold of *p* < 0.001, to assess the one-sample and two-sample *t* tests derived from the target independent components (DMN, left FPCN and right FPCN). Multiple comparison correction was applied at the cluster level using family-wise error (FWE) and false-discovery rate (FDR) corrections. Parametric statistics were chosen as the whole-brain connectivity values followed a normal distribution after preprocessing and denoising [45].

### Seed-based connectivity analyses

In order to investigate the functional connectivity of the anterior and posterior portions of the PHG in both hemispheres with the rest of the brain, whole-brain seed–based connectivity analyses (SBA) were performed. The anatomical parcellation derived from the Harvard–Oxford brain atlas, integrated within the CONN toolbox, was used to define the seed regions of interest ([41]; see the CONN website for more information about this atlas). This parcellation scheme is consistent with the proposed anatomical division of the PHG mentioned in the introduction, in which the aPHG corresponds to the perirhinal/entorhinal cortex and the pPHG corresponds to the parahippocampal cortex [16, 18].

A general linear model (GLM) was used with a hemodynamic response function (HRF) weighted to the first level, and the standard bivariate correlation measure was used for functional connectivity analyses.

Individual seed-to-voxel functional connectivity maps were generated for each participant and seed. The correlations of the BOLD signal along the time series were computed across all voxels within each region of interest (ROI) and the rest of the brain. Fisher’s *z* transformation was subsequently applied to the correlation coefficients for each pair of sources (seed and every brain voxel), resulting in *z* score connectivity maps. This transformation ensures that the connectivity values are normally distributed, as required in SBA. The resulting *z* score connectivity maps of each seed and participant were used for the second-level analyses [41].

Considering the potential impact of atrophy on functional connectivity by inducing artifacts, it was deemed necessary to account for this effect in the SBA considering that the PHG is a key region within the MTL that demonstrates early atrophy in the AD spectrum [19, 46]. To address the potential confounding influence of structural alterations on functional connectivity measures, the volume of gray matter in the PHG seed regions was included as a covariate in all SBA.

The significance level was set at *p* < 0.05 for the cluster level, in conjunction with an uncorrected voxel-level threshold of *p* < 0.001, to assess the one-sample and two-sample *t* tests derived from the seed-to-voxel maps of the target ROIs (left aPHG/pPHG and right aPHG/pPHG). Multiple comparison correction was applied at the cluster level by using FWE and FDR corrections. Parametric statistics were used as the *z* scores conforming the connectivity maps in the second-level SBA were normally distributed [41].

### Post hoc ROI-to-ROI analyses

Post hoc analyses, aimed at gaining further insights into the results obtained from the SBA and its relationship with the DMN, involved extracting values of the functional connectivity between the region that exhibited the greatest differences in the SBA (left pPHG) and four key nodes of the DMN of the network atlas included in CONN (i.e., the precuneus cortex, right and left lateral parietal cortex, and medial prefrontal cortex seeds derived from an ICA of a Human Connectome Project data set (*n* = 497); see the CONN website for more information on this atlas).

To this end, we used the Rex toolbox, implemented in the CONN toolbox, to extract the individual *z* scores for each of the participants and each of the DMN seeds from the connectivity maps of the left pPHG [41].

In order to determine the connectivity profiles of each of the groups in the functional connectivity between the left pPHG and the DMN nodes, ANCOVA corrected for multiple comparisons with Bonferroni correction, including years of formal education and the volume of gray matter of the left pPHG as covariates, was conducted using IBM SPSS version 25.0 statistical software (https://www.ibm.com/es-es/products/spss-statistics). The normal distribution of the connectivity values was tested using the Kolmogorov–Smirnov test. Parametric statistics were used as *z* scores derived from the seeds were normally distributed [41].

### Post hoc intra/inter-network connectivity and moderation analyses

In the post hoc analyses, we specifically examined the connectivity in the DMN and the salience network (SAL) (i.e., intra-network connectivity) and also the connectivity between these two networks (i.e., inter-network connectivity). The rationale for conducting these types of analyses stemmed from the results observed in the whole-brain SBA, which highlighted multiple significant findings within the context of both the DMN and the connectivity between the DMN-SAL. Consequently, these analyses were conducted to determine whether the observed differences are restricted to the DMN-SAL inter-network connectivity or also compromised the intra-network connectivity of the SAL. Additionally, by providing a unified measure of within- and between-network connectivity, these analyses make subsequent moderation analyses operationally more feasible, as they yield a single metric for each network and their interactions. To calculate the connectivity within the DMN/SAL and between the DMN/SAL, we used the seeds included in the CONN network atlas. Additionally, for the DMN, we included bilateral seeds from the aPHG and pPHG. The ROI-to-ROI matrices, containing Fisher’s *z*-transformed connectivity values, were used to determine the intra/inter-connectivity of the DMN and SAL following a method similar to [47]. ANCOVA corrected for multiple comparisons with Bonferroni correction was performed employing years of formal education as covariable using IBM SPSS version 25.0 statistical software. The normal distribution of the connectivity values was tested using the Kolmogorov–Smirnov test.

Moderation analyses were then performed using neuropsychological measures to explore how the connectivity metrics interact with cognitive performance across the entire sample, further elucidating the role of network dynamics in cognitive outcomes. The moderation analyses also included the observed results for the left FPCN in frontal and posterior regions, as identified in the MCI subgroups relative to the control group in the ICA. These analyses were performed using “lavaan” in RStudio [48]. A bootstrapping procedure of 5000 samples was employed.

We focused on three specific moderation models to explore how network dynamics interact with cognitive outcomes. The first model examined the interaction between SAL-DMN connectivity and DMN connectivity on memory performance, controlling for age and years of education. This approach was driven by the differences observed across groups in the network dynamics in the SBC and the established association between DMN changes and episodic memory [49], as well as evidence linking the degradation of the DMN/SAL network connectivity linked to amyloid burden in symptomatic individuals [50].

The second model was used to assess the influence of the interaction between left FPCN connectivity in posterior regions and years of education on executive function, with age included as a covariate. The FPCN’s role in executive control, particularly in posterior regions, supports the relevance of this analysis [13, 51, 52].

The third model was used to explore how the interaction between FPCN connectivity and years of education affects language performance, while accounting for age as a covariate. This model was justified by the specialization of the FPCN in frontal regions associated with language production [42, 51].

In the moderation models involving the FPCN, years of formal education were included as a moderating variable, as previous research has shown that education is linked to changes in the functional connectivity of similar FPCN regions in healthy aging [53].

## Results

### Neuropsychological and behavioral assessment

The control group performed better than the sd-aMCI group in all memory tests, as well as in one executive function test (phonological fluency) and one language test (Boston naming test) (see Table 1). The control and sd-aMCI groups obtained higher scores than the md-aMCI group in various cognitive domains (memory, executive function, attention, and language).

No significant differences were observed between the groups in the Lawton and Brody Scale, indicating that functional abilities related to activities of daily living were relatively consistent across the groups. Moreover, no significant differences in the presence of depressive symptoms were noted across the groups.

Importantly, given the significant differences in years of education between the groups, this factor was included as a covariate in all second-level analyses.

### Volumetric ROI assessment

In relation to the PHG ROI gray matter volume, significant differences emerged for the pPHG ROIs (left and right) between the groups: the volume was lower in the md-aMCI group than in both the control and sd-aMCI groups. The aPHG seeds followed a similar trend, although the differences were not statistically significant (see Table 1).

### Independent component analysis

**Fig. 2:**
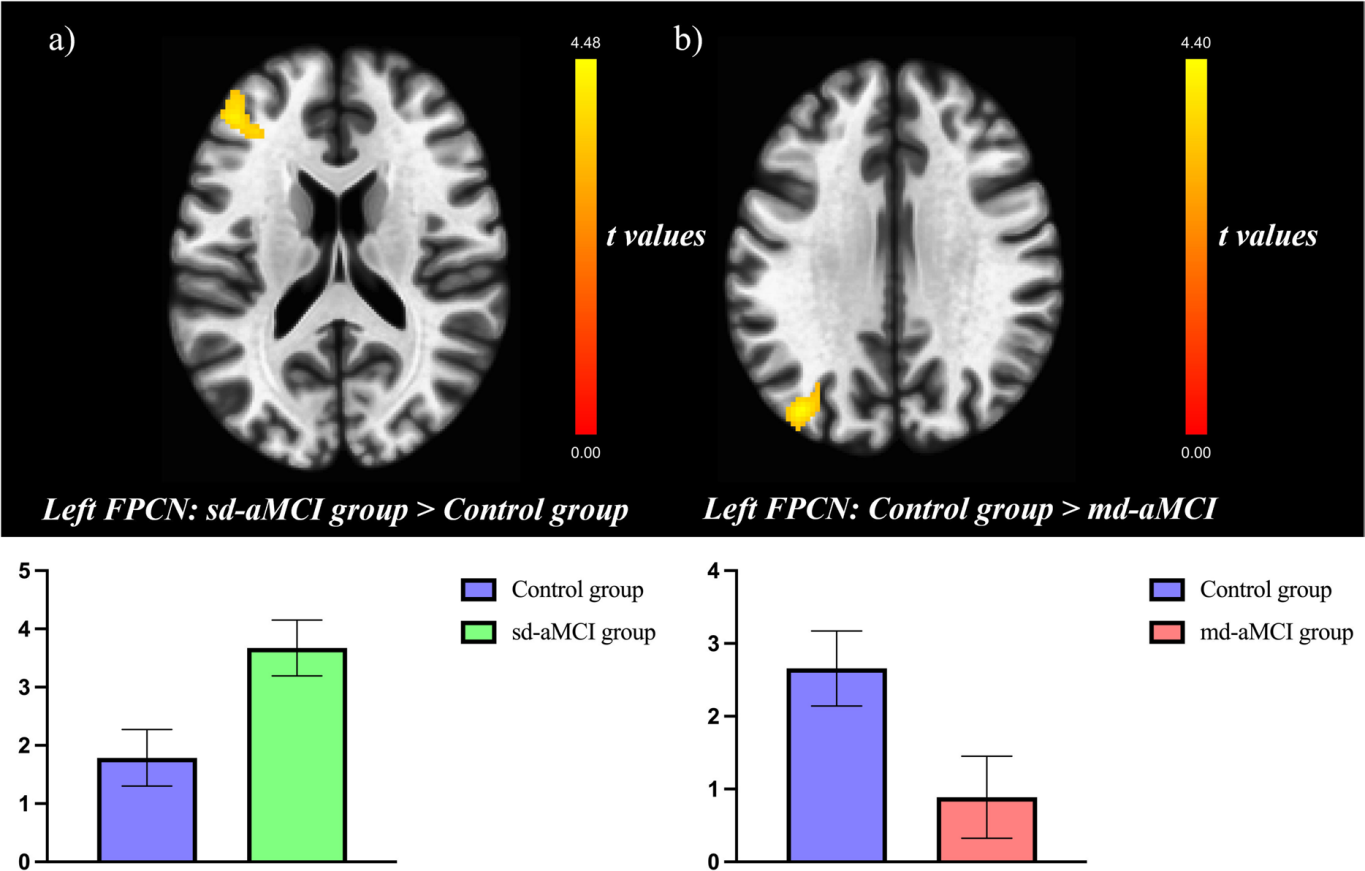
Significant differences in brain connectivity observed by ICA of the left FPCN, colored bars represent *t* scores of the clusters derived from the group analyses of connectivity maps, serving as a legend for the connectivity maps shown on the brain surfaces (top). Mean activation values of the significant clusters, the error bars represent the 95% confidence interval (bottom). Higher left FPCN connectivity in the sd-aMCI than in controls (**a**). Lower left FPCN connectivity in the md-aMCI than in the control group (**b**). Results are significant at *p* < 0.05 FWE and FDR cluster-corrected in a combination with a threshold of *p* < 0.001 at the uncorrected voxel level for the sd-aMCI vs. control group comparison (**a**), while the md-aMCI vs. control group comparison only survived the FDR cluster correction (**b**)

For the left FPCN, the ICA showed a group-level effect, specifically in a cluster comprising the pars triangularis of the left inferior frontal gyrus and the left middle frontal gyrus (see Online Resources 2). For the other components evaluated (DMN and right FPCN), the *F* test did not reveal any group effects. Post hoc comparisons showed that relative to the control group, the sd-aMCI group exhibited higher functional connectivity in that cluster, while the md-aMCI group showed lower functional connectivity in a cluster comprised by the left middle occipital gyrus (only significant at cluster-level FDR correction). No further differences were observed (see Table 2 and Fig. 2).

**Table 2:** Significant ICA results across groups

	Brain region	Cluster size	# voxels in specific region (% overlap)	L/R	MNI coordinates (x, y, z)			Statistic *t*
Left FPCN	sd-aMCI > control group							
	Middle frontal gyrus (dorsolateral part)	313	165 (4)	L	− 44	44	16	4.48
	Inferior frontal gyrus (*pars triangularis*)		140 (6)	L				
	Control group > md-aMCI							
	Middle occipital gyrus	161*	132 (4)	L	− 38	− 78	32	4.40

*L/R* Left or right hemisphere; *MNI* Montreal Neurological Institute coordinates. Results are significant at *p* < 0.05 FWE and FDR cluster-corrected in a combination with a threshold of *p* < 0.001 at the uncorrected voxel level. Only brain regions with > 1% cluster overlap are included

^*^Only significant after FDR cluster-correction

### Seed-based connectivity analyses

The results of the one-sample *t* test of the SBA are shown in Online Resources 3. The SBA only showed a group-level effect for the left parahippocampal seeds (aPHG and pPHG) in the *F* test (see Online Resources 4 and 5 respectively).

**Fig. 3:**
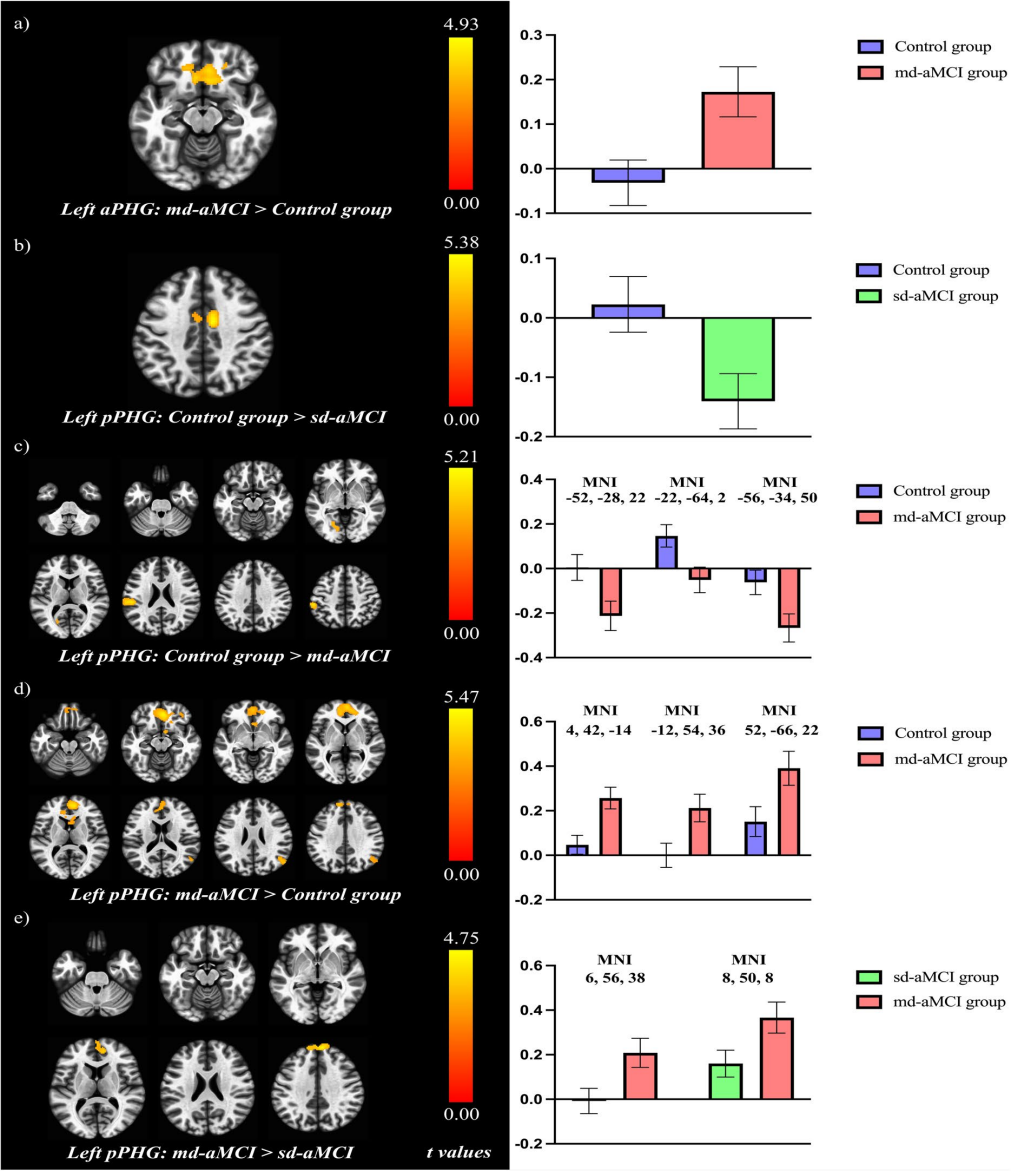
Significant differences in brain connectivity obtained through the SBA of the left aPHG (**a**) and pPHG (**b**, **c, d**, and **e**), the colored bars represent *t* scores of the clusters derived from the group analyses of connectivity maps, serving as a legend for the connectivity maps shown on the brain surfaces (left). Mean *z* scores values derived from the significant clusters, the error bars represent the 95% confidence interval. For the graphs with more than one cluster peak MNI coordinates are specify (right). Results are significant at *p* < 0.05 FWE and FDR cluster-corrected in a combination with a threshold of *p* < 0.001 at the uncorrected voxel level

The analyses revealed a group effect on aPHG connectivity, with a cluster in the orbitofrontal cortex (rectus gyrus, medial-orbital frontal gyrus, medial orbital gyrus, olfactory cortex, anterior orbital gyrus, and posterior orbital gyrus) extending to the subgenual cortex in the anterior cingulate cortex. Post hoc comparisons revealed higher functional connectivity within a cluster mainly located in orbitofrontal regions in the md-aMCI group respected the control group (see Table 3 and Fig. 3a).

**Table 3:** Brain regions showing significant group differences in the SBA of the left aPHG and pPHG

	Brain region	Cluster size	# voxels in specific region (% overlap)	L/R	MNI coordinates (x, y, z)			Statistic *t*	
Left aPHG	md-aMCI > control group								
	Gyrus rectus	920	295 (40)	R	8	36	− 18	4.93	
	Gyrus rectus		169 (20)	L					
	Superior frontal gyrus (medial orbital part)		95 (11)	R					
	Medial orbital gyrus		68 (11)	R					
	Superior frontal gyrus (medial orbital part)		56 (8)	L					
	Medial orbital gyrus		39 (7)	L					
	Anterior orbital gyrus		34 (8)	L					
	Olfactory cortex		31 (11)	R					
	Anterior cingulate cortex (subgenual part)		28 (17)	L					
	Olfactory cortex		17 (6)	L					
	Anterior cingulate cortex (subgenual part)		14 (11)	R					
	Nucleus accumbens		3 (2)	R					
Left pPHG	Control group > sd-aMCI								
	Middle cingulate and paracingulate gyri	261*	169 (8)	R	10	− 4	40	5.38	
	Middle cingulate and paracingulate gyri		70 (4)	L					
	Control group > md-aMCI								
	Supramarginal gyri	510*	262 (21)	L	− 52	− 28	22	5.21	
	Postcentral gyrus		102 (3)	L					
	Superior temporal gyrus		69 (3)	L					
	Rolandic operculum		37 (4)	L					
	Calcarine	280	112 (5)	L	− 22	− 64	2	4.78	
	Lingual gyrus		111 (5)	L					
	Inferior parietal gyrus	214	142 (6)	L	− 56	− 34	50	4.69	
	md-aMCI > control group								
	Superior frontal gyrus (medial part)	2893*	350 (16)	R	4	42	− 14	5.47	
	Superior frontal gyrus (medial part)		340 (11)	L					
	Superior frontal gyrus (medial orbital part)		340 (47)	L					
	Superior frontal gyrus (medial orbital part)		336 (39)	R					
	Anterior cingulate cortex (pregenual part)		324 (52)	L					
	Gyrus rectus		200 (23)	L					
	Gyrus rectus		188 (25)	R					
	Anterior cingulate cortex (pregenual part)		155 (24)	R					
	Olfactory cortex		31 (11)	R					
	Anterior orbital gyrus		24 (4)	R					
	Nucleus accumbens		20 (14)	R					
	Medial orbital gyrus		19 (3)	L					
	Medial orbital gyrus		18 (3)	R					
	Inferior frontal gyrus (*pars orbitalis*)		16 (2)	R					
	Olfactory cortex		15 (5)	L					
	Anterior cingulate cortex (subgenual part)		11 (7)	L					
	Anterior cingulate cortex (subgenual part)		9 (7)	R					
	Superior frontal gyrus (dorsolateral part)	410	174 (4)	L	− 12	54	36	4.48	
	Superior frontal gyrus (medial part)		145 (5)	L					
	Superior frontal gyrus (medial part)		69 (3)	R					
	Angular gyrus	336	207 (12)	R	52	− 66	22	4.31	
	Middle temporal gyrus		68 (2)	R					
	Middle occipital gyrus		54 (3)	R					
	md-aMCI > sd-aMCI								
	Superior frontal gyrus (medial part)	333	118 (4)	L	6	56	38	4.75	
	Superior frontal gyrus (medial part)		100 (5)	R					
	Superior frontal gyrus (medial part)	282	141 (7)	R	8	50	8	4.40	
	Superior frontal gyrus (medial part)		83 (3)	L					
	Anterior cingulate cortex (pregenual part)		41 (6)	R					

*L/R* Left or right hemisphere, *MNI* Montreal Neurological Institute coordinates. Results are significant at *p* < 0.05 FWE and FDR cluster-corrected in a combination with a threshold of *p* < 0.001 at the uncorrected voxel level. Only brain regions with > 1% cluster overlap are included

^*^Also significant at peak p-FWE level

A group effect was observed for the connectivity of the pPHG seed with three different clusters (see Online Resources 5), two located in frontal regions (mainly the superior frontal gyrus and anterior cingulate cortex) and the other in parieto-temporal regions (supramarginal gyrus, postcentral gyrus, superior temporal gyrus, and the Rolandic operculum).

The pPHG post hoc comparisons revealed higher functional connectivity between this seed and the left and right middle cingulate cortex in the control group than in the sd-aMCI group. This effect arose from negative connectivity values in the sd-aMCI group and positive connectivity values in the control group (see Table 3 and Fig. 3b).

Functional connectivity between the pPHG and three clusters was higher in the control group than in the md-aMCI group. Two of the clusters were mainly located in centro-parietal regions (parietal operculum, anterior/posterior supramarginal gyrus, central opercular cortex, postcentral gyrus, inferior parietal gyrus), and the other was located in temporo-occipital regions (intracalcarine cortex and lingual gyrus) of the left hemisphere (see Table 3 and Fig. 3c). The enhanced connectivity between the pPHG and centro-parietal regions was explained by negative connectivity in the md-aMCI, while the control group exhibited connectivity close to zero (see Fig. 3c). However, the increased connectivity between the pPHG and the cluster located in temporo-occipital regions is explained by the positive connectivity in the control group and by the negative connectivity in the md-aMCI group (see Fig. 3c). In addition, the higher connectivity in the md-aMCI group than in the control group was observed in various regions in the frontal (mid and superior frontal gyrus, anterior cingulate cortex, and orbitofrontal regions) and parietal lobes (see Table 3 and Fig. 3d).

Finally, comparison of the sd-aMCI and md-aMCI groups in relation to pPHG connectivity revealed an increase in connectivity between this area and frontal regions (including the medial section of the superior frontal gyrus, dorsolateral part of the superior frontal gyrus and the anterior cingulate cortex) in the md-aMCI group relative to the sd-aMCI group (see Table 3 and Fig. 3e).

### Post hoc ROI-to-ROI analyses

Regarding the post hoc analyses of region-of-interest ROI-to-ROI connectivity between the nodes of the DMN and the region showing the maximum between-group differences (left pPHG), a group effect was observed for the connectivity between the pPHG seed and the mPFC as well as the right lateral parietal cortex (RLPC).

For both the pPHG-mPFC and pPHG-RLPC connectivity, multiple comparisons revealed significantly higher levels of connectivity in the md-aMCI group than in both the control group and the sd-aMCI group (see Online Resources 6 and 7).

### Post hoc intra/inter-network connectivity and moderation analyses

Regarding the intrinsic connectivity within the DMN and SAL, a significant effect across groups was observed only for the intrinsic connectivity of the DMN. Specifically, the md-aMCI group showed greater functional connectivity within the DMN than the control group (*p* = 0.019). However, when the connectivity was analyzed using all network nodes simultaneously, and although the sd-aMCI group exhibited lower connectivity than the md-aMCI group, the difference between the sd-aMCI and md-aMCI groups was not statistically significant (*p* = 0.183) (see Online Resources 8).

Regarding the between-network connectivity (SAL-DMN), a significant effect was observed. Specifically, the control group exhibited higher connectivity levels than the md-aMCI group (*p* = 0.013). Additionally, the sd-aMCI group showed higher connectivity levels than the md-aMCI group, but these differences were not statistically significant (*p* = 0.084) (see Online Resources 8).

The first moderation model showed a significant positive effect of SAL-DMN connectivity on memory scores (5.696, 95% CI [1.868, 10.262], *p* = 0.006). Conversely, a significant negative effect was observed for DMN connectivity on memory scores (− 2.907, 95% CI [− 5.520, − 0.420], *p* = 0.026). A significant effect of the interaction between SAL-DMN connectivity and DMN connectivity was also identified (− 13.406, 95% CI [− 23.151, − 2.855], *p* = 0.007). These findings suggest that higher SAL-DMN connectivity is associated with better memory performance, whereas increased DMN connectivity alone is linked to poorer memory outcomes. The SAL-DMN effect was significantly moderated by DMN connectivity, so that higher DMN connectivity reduced the positive impact of SAL-DMN connectivity on memory performance. Additionally, regression analysis showed a significant negative link between DMN connectivity and SAL-DMN connectivity (− 0.727, 95% CI [− 0.899, − 0.554], *p* < 0.001), indicating that as SAL-DMN connectivity increases, DMN connectivity decreases.

The second moderation model showed a significant positive effect of the connectivity derived from left FPCN connectivity in posterior regions on executive function scores (0.110, 95% CI [− 0.004, 0.183], *p* = 0.020). Furthermore, the years of education factor significantly predicted executive function (0.319, 95% CI [0.223, 0.532], *p* < 0.001). However, no significant interaction between left FPCN connectivity in posterior regions and years of education on executive function was observed (− 0.031, 95% CI [− 0.188, 0.059], *p* = 0.584). Although a significant positive effect of left FPCN connectivity in posterior regions on executive function was detected, the confidence interval includes zero (95% CI [− 0.004, 0.183]), suggesting that the effect size may be small and should be interpreted with caution.

Finally, a significant positive effect of left FPCN connectivity in frontal regions on language scores was observed (0.149, 95% CI [0.005, 0.302], *p* = 0.047). While statistically significant, the lower boundary of the confidence interval is close to zero, suggesting a possibly small effect and warranting careful interpretation. The analysis also revealed a significant positive effect of years of education (0.505, 95% CI [0.359, 0.675], *p* < 0.001) on language scores. The interaction effect between left FPCN connectivity in frontal regions and years of education was not statistically significant (− 0.142, 95% CI [− 0.308, 0.016], *p* = 0.083).

## Discussion

In this study, we aimed to improve the characterization of the functional correlates of two distinct aMCI subgroups (sd-aMCI and md-aMCI), by exploring the resting-state brain connectivity of two networks, the DMN and the FPCN, which potentially reflect compensatory and deteriorative processes in individuals with aMCI [11, 21–23]. We used ICA to investigate the intrinsic connectivity of these networks across the three groups. In addition, we performed a whole-brain SBA, considering the bilateral aPHG and pPHG as regions of interest. To date, very few studies have considered aMCI subtypes [6, 28]. Moreover, we conducted post hoc analyses of the putative causal linkages between functional connectivity and neuropsychological variables, aiming to better understand how these networks are associated with cognitive performance.

Regarding the ICA results, we observed a group-level effect for the left FPCN. Connectivity was higher in the sd-aMCI group than in the control group, within a cluster spanning from the left inferior frontal gyrus (pars triangularis) to the left middle frontal gyrus. By contrast, connectivity was lower in the md-aMCI group than in the control group in a cluster mainly involving the middle occipital gyrus.

Numerous studies have highlighted the role of FPCN connectivity, particularly its frontal regions, as a proxy for cognitive reserve/compensation in old adults with and without MCI [53, 54]. The higher FPCN connectivity in the sd-aMCI group than in the control group, specifically in the ventrolateral prefrontal cortex (pars triangularis) and dorsolateral prefrontal cortex (middle frontal gyrus), suggests functional reorganization and recruitment of additional neural resources in these participants. This could be explained by the scaffolding theory of aging and cognition, which predicts increased recruitment of frontal regions as functional compensation in the aging process [30, 54–56]. Moreover, consistent with this interpretation, the moderation analyses revealed a significant positive effect of left FPCN connectivity in frontal regions on language scores, highlighting the functional importance of this network. Language scores were used in this model because the frontal cluster of the left FPCN overlapped partly with Broca’s area, a region that is important for language production. Broca’s area also plays a key role in the phonological loop of working memory, facilitating the rehearsal of verbal information [42, 51]. Interestingly, the moderation analyses evidenced that education has a direct effect on language scores but does not interact with the connectivity of frontal regions in the left FPCN. In contrast to observations made in healthy aging populations, this suggests that cognitive reserve associated with years of formal education may depend on different brain regions in the context of MCI and the compensatory effect of the frontal regions of the left FPCN seems to be independent of cognitive reserve [53]. However, these findings should be interpreted with caution due to the limited statistical power resulting from the small sample size.

Connectivity was lower in the md-aMCI group than in the control group within this network in parieto-occipital regions. Similar results were reported by Li et al. [52] who observed lower connectivity in the middle occipital and angular gyrus in MCI participants who converted to AD dementia (observed at follow-up) than in non-converters, which was interpreted as explaining damage in multiple cognitive domains in patients converting to AD. Consistent with the findings of the aforementioned study, the moderation model conducted in our work with the connectivity of posterior regions of the left FPCN showed a significant positive effect of left FPCN connectivity on executive function scores, while the interaction with education was not significant. Education appears to have a different effect in this sample, which includes MCI participants, than in healthy aging population [53], with a positive effect on executive function but independent of the connectivity in these posterior regions of the left FPCN.

The ICA did not reveal any between-group differences in connectivity within the DMN; however, SBA of the parahippocampal ROIs (aPHG and pPHG), a hub that links the MTL and the cortical nodes of the DMN, showed significant differences between the groups in the left hemisphere ROIs. Unsurprisingly, significant results were only observed for the left hemisphere ROIs as numerous studies conducted with various types of neuroimaging have consistently reported more severe deterioration in regions of the left MTL in the AD continuum [57–59].

SBA provided evidence of significantly higher functional connectivity between the aPHG and orbitofrontal region in the md-aMCI group than in the control group. The aPHG (including the perirhinal and entorhinal cortex) belongs to the anterior-temporal (AT) memory subsystem within the medial temporal lobe memory system [16]. This result can possibly be attributed to neural dysregulation caused by the degeneration of key MTL regions in md-aMCI participants. The entorhinal cortex, which together with the hippocampus forms the perforant pathway, is known to have an inhibitory feedback mechanism (via bidirectional projection of GABAergic neurons), and impairment of the GABAergic circuits in the MTL has been observed in early AD [60]. Impairment of key regions of the MTL, as a result of underlying pathological processes, may result in the disinhibition of MTL brain regions, including the hippocampus, PHG, and other brain regions connected to MTL. This hypothesis provides a potential explanation for the disinhibition observed in MTL structures and directly connected cortical regions, including those within the DMN observed in this and previous studies on the AD continuum, in which increased functional connectivity within the DMN and its MTL sub-system was observed [22, 23, 26].

Regarding the pPHG seed, group effects were observed in frontal and parieto-temporal regions. Multiple comparisons revealed higher levels of connectivity with a cluster located in the middle cingulate in the control group than in the sd-aMCI group, and with central-parietal and temporo-occipital areas in the md-aMCI groups. In both cases, these structures form part of a different functional network named the SAL network [61]. The SAL network is known to be responsible for detecting salient stimuli and facilitating transitions between the DMN and the FPCN [62, 63]. The post hoc analyses of intrinsic SAL connectivity and SAL-DMN connectivity revealed significant group differences only for the inter-network connectivity, with SAL connectivity itself remaining preserved. However, in these analyses, only differences between the control group and the md-aMCI group were observed. This supports the idea that changes in the AD spectrum primarily originate within the DMN, affecting its connectivity, disrupting cerebral homeostasis and altering interactions between the DMN and other networks, such as the SAL. Remarkably, one study reported that participants diagnosed with AD dementia exhibited a notable decrease in effective connectivity between the SAL and DMN relative to the control group [64]. Moreover, another study found that deterioration of the functional relationship between the SAL and DMN is related to amyloid burden in symptomatic individuals [50]. The differences in functional connectivity between the DMN, evoked from the left pPHG seed, and SAL regions in both subtypes of aMCI participants than in the controls, suggest some disruption in the connections between these networks. These changes may affect the functional dynamics between the DMN and the FPCN [62, 65] and thus significantly contribute to cognitive decline in the aMCI participants in our study. However, md-aMCI participants exhibited a different pattern of functional connectivity than the other groups. Specifically, they exhibited a greater increase in pPHG connectivity with frontal and parietal regions of the DMN than in the control group as well as higher functional connectivity of the pPHG with frontal DMN regions than in the sd-aMCI group in the seed-to-voxel analyses. Moreover, post hoc ROI-to-ROI analyses conducted using the main nodes of the DMN and the pPHG confirmed this hyperconnectivity pattern in the md-aMCI group relative to both the control and sd-aMCI groups (see Online Resources 6 and 7). However, when intrinsic DMN connectivity was calculated using bilateral PHG seeds (anterior and posterior) along with the cortical DMN seeds, significant differences were only observed between the md-aMCI group and controls, and no significant differences between the sd-aMCI and md-aMCI groups were detected. This finding may support the concept of a continuum in MCI subtypes. Hyperconnectivity of the MTL memory system and other cortical regions of the DMN has previously observed in patients with MCI, patients with subjective cognitive decline and in APOEε4 carriers [22–26, 61]. Most studies attribute the DMN hyperconnectivity to compensatory phenomena [22–25, 61]. Given the poorer performance of the md-aMCI participants in neuropsychological tests and the lack of hyperconnectivity in sd-aMCI participants and the control group in our study, we are hesitant to attribute this to a typical compensatory phenomenon. Furthermore, the moderation analysis revealed that hyperconnectivity within the DMN is associated with poorer memory scores, while increased connectivity between the SAL and DMN is linked to better memory performance. Notably, the effect of the interaction between SAL-DMN connectivity and DMN connectivity was significant, indicating that the balance between these networks plays a crucial role in modulating cognitive outcomes, particularly memory. We propose that the hyperconnectivity arises directly from the underlying MTL pathology, leading to pathological disinhibition in these regions and other cortical DMN regions. A similar interpretation was also made by [26], who pharmacologically reduced hippocampal hyperactivation in aMCI patients, leading to better performance of the scanner task. Consistent with this disinhibition hypothesis, the linear regression analysis revealed an inverse relationship between intrinsic DMN connectivity and the connectivity between the SAL and DMN. Greater connectivity within the DMN seems to isolate the network, increasing its dominance and reducing the connectivity between the SAL and DMN.

The present study is not without limitations. First, the cross-sectional nature of the study only enables suggestive rather than confirmatory conclusions to be reached. The lack of available biological markers for tau and beta-amyloid, with only neuropsychological diagnosis being applied, is another limitation. Future studies should consider using a longitudinal approach to examine the subtypes of aMCI, increasing the statistical power and establishing the relationship between connectivity and levels of pathological protein aggregates. This would enable more robust conclusions to be reached.

In summary, the present study contributed to characterizing the neurophysiological correlates of aMCI subtypes, revealing that, consistent with the clinical symptoms (impairment of cognitive domains), sd-aMCI and md-aMCI participants exhibited gradual and distinctive functional connectivity patterns, while the sd-aMCI group showed evidence of compensatory (higher FPCN than the control group) and dysfunctional (lower pPHG connectivity with structures of the SAL network) activity, md-aMCI participants exhibited a more disrupted connectivity pattern (lower FPCN connectivity than in the control group, lower pPHG connectivity with structures of the SAL network than in the control group, higher pPHG connectivity with structures of the DMN than in both control and sd-aMCI participants), potentially contributing to more widespread and severe cognitive deficits in this subgroup. Indeed, post hoc analysis of the results obtained for the left FPCN connectivity in anterior and posterior brain regions revealed a positive relationship with language scores and executive function, respectively, indicating a potential compensatory effect in the context of MCI that appears to be independent of the influence of formal years of education. Moreover, DMN hyperconnectivity is linked to poorer memory performance, while stronger SAL-DMN connectivity is associated with better memory outcomes. Additionally, linear regression showed that greater DMN connectivity corresponds to reduced SAL-DMN connectivity, highlighting the importance of network balance in cognitive function. The confluence of gradual and qualitative modifications further substantiates the distinction between aMCI subtypes and may explain the different effects reported in previous studies, providing potential biomarkers for diagnosis and indicating possible therapeutic targets aimed at improving neural activity in these populations (e.g., using non-invasive brain stimulation).

## Supplementary Information

Below is the link to the electronic supplementary material.

Supplementary file1 (DOCX 66 KB)

Supplementary file2 (DOCX 17 KB)

Supplementary file3 (PNG 1.56 MB)

High resolution image (TIF 879 KB)

Supplementary file4 (DOCX 17 KB)

Supplementary file6 (DOCX 42 KB)

Supplementary file7 (DOCX 15 KB)

Supplementary file3 (PNG 89.9 KB)

High resolution image (TIF 380 KB)

Supplementary file8 (DOCX 17 KB)

## Data Availability

Data will be made available on request.
