# Supplementary material for: Compensation versus deterioration across functional networks in amnestic mild cognitive impairment subtypes
Source: GeroScience. 2024 Oct 5;47(2):1805–22. doi: 10.1007/s11357-024-01369-9 (PMC11978594; doi:10.1007/s11357-024-01369-9)
Supplement: Supplementary file 1 — Supplementary file1 (DOCX 66 KB) [file 11357_2024_1369_MOESM1_ESM.docx]

**Appendix A1**

**Data pre-processing and functional connectivity analysis**

The structural and functional images underwent preprocessing and analysis using the CONN 19c toolbox [1] and SPM12 toolbox (Wellcome Department of Imaging Neuroscience, London, UK; <http://www.fil.ion.ucl.ac.uk/spm/>) implemented in Matlab R2019a.

For preprocessing, we followed the default pipeline of the CONN toolbox, which involved several steps. Functional realignment and unwarping were performed, followed by functional centering of the image to (0, 0, 0) coordinates. Slice-timing correction was applied, and functional outlier detection was conducted. The images were simultaneously subjected to functional direct segmentation and normalization to the MNI space. Structural images were also centered to (0, 0, 0) coordinates and underwent simultaneous structural segmentation and normalization to MNI space. Finally, spatial smoothing with an 8 mm FWHM kernel was applied.

To address physiological sources of noise, we utilized the anatomical component-based noise correction procedure (aCompCor) implemented in the CONN toolbox [2]. This approach extracted white matter and cerebrospinal fluid noise components, primarily controlling for cardiac pulsations and respiration-induced modulations of the main magnetic field. We selected aCompCor due to its demonstrated specificity and sensitivity for positive correlations while addressing negative correlations (anti-correlations) [3]. The Artifact Detection Toolbox (ART) [1, 4] was used for quantifying participant motion and identifying outlier scans. The ART parameters were set to the 97th percentile, with the mean global signal deviation threshold at z = ±5 and the participant motion threshold at 0.9 mm. All study participants exhibited more than 5 minutes of signal after denoising, which contributed to ensuring the robustness of the signal [5].

Linear regression of potential confounds and a temporal band-pass filter of 0.008–0.09 Hz were applied to the data to exclude signal frequencies outside the expected BOLD range, reduce the impact of the confound effects [1]. No significant differences in movement parameters measured by CONN nor in the number of outlier scans detected by ART were observed between groups.

Two modalities of resting-state functional connectivity analysis were employed to investigate the DMN and the FPCN. Firstly, ICA was conducted to assess the intrinsic connectivity within these target networks [6]. Furthermore, to investigate the functional connectivity of the PHG, a seed-to-voxel analysis was performed using the parcellations of the anterior and posterior PHG derived from the Harvard-Oxford atlas included in CONN [1, 6].

The gray matter volumes of each PHG seed used in the analyses were extracted for each participant. This process involved utilizing the gray matter T1 segmentations estimated by SPM12 during preprocessing. These segmentations were then intersected with the Oxford-Harvard brain atlas using FSL [7] to obtain the grey matter volume for each seed and participant. Subsequently, the seed volumes were adjusted by the participants' total intracranial volume (ITV) to account for individual variations in brain size by using the formula, adjusted_volume = volume_observed – b * (TIV – mean_TIV) where mean_TIV is the average TIV of all participants and b is the coefficient of regression between the volume observed and the TIV [8].

**References**

1. Whitfield-Gabrieli S, Nieto-Castanon A (2012) Conn : A Functional Connectivity Toolbox for Correlated and Anticorrelated Brain Networks. Brain Connect 2:125–141. https://doi.org/10.1089/brain.2012.0073

2. Behzadi Y, Restom K, Liau J, Liu TT (2007) A component based noise correction method (CompCor) for BOLD and perfusion based fMRI. Neuroimage 37:90–101. https://doi.org/10.1016/j.neuroimage.2007.04.042

3. Chai XJ, Castañón AN, Öngür D, Whitfield-Gabrieli S (2012) Anticorrelations in resting state networks without global signal regression. Neuroimage 59:1420–1428. https://doi.org/10.1016/j.neuroimage.2011.08.048

4. Shirer WR, Jiang H, Price CM, et al (2015) Optimization of rs-fMRI Pre-processing for Enhanced Signal-Noise Separation, Test-Retest Reliability, and Group Discrimination. Neuroimage 117:67–79. https://doi.org/10.1016/j.neuroimage.2015.05.015

5. Van Dijk KRA, Hedden T, Venkataraman A, et al (2010) Intrinsic functional connectivity as a tool for human connectomics: Theory, properties, and optimization. J Neurophysiol 103:297–321. https://doi.org/10.1152/JN.00783.2009/ASSET/IMAGES/LARGE/Z9K0011098670015.JPEG

6. Yang J, Gohel S, Vachha B (2020) Current methods and new directions in resting state fMRI. Clin Imaging 65:47–53. https://doi.org/10.1016/j.clinimag.2020.04.004

7. Smith SM, Jenkinson M, Woolrich MW, et al (2004) Advances in functional and structural MR image analysis and implementation as FSL. Neuroimage 23:S208–S219. https://doi.org/10.1016/J.NEUROIMAGE.2004.07.051

8. Voevodskaya O, Simmons A, Nordenskjöld R, et al (2014) The effects of intracranial volume adjustment approaches on multiple regional MRI volumes in healthy aging and Alzheimer’s disease. Front Aging Neurosci 6:264. https://doi.org/10.3389/fnagi.2014.00264
