# Supplementary material for: Compensation versus deterioration across functional networks in amnestic mild cognitive impairment subtypes
Source: GeroScience. 2024 Oct 5;47(2):1805–22. doi: 10.1007/s11357-024-01369-9 (PMC11978594; doi:10.1007/s11357-024-01369-9)
Supplement: Supplementary file 2 — Supplementary file2 (DOCX 17 KB) [file 11357_2024_1369_MOESM2_ESM.docx]

| **Table 1.** Group effect obtained across groups for the ICA results. | | | | | | | | | |
| --- | --- | --- | --- | --- | --- | --- | --- | --- | --- |
|  | **Brain region** | **Cluster size** | **# voxels in specific region**  **(% overlap)** | **L/R** | **MNI Coordinates (x,y,z)** | | | **Statistic** | |
|  |  |  |  |  |  | | | **F** | |
| **Left FPCN** | **Group effect** |  |  |  |  |  |  |  |  |
|  | Inferior frontal gyrus (*pars triangularis*) | 150 | 75 (3) | L | -36 | 32 | 12 | 10.55 | |
|  |  |  |  |  |  |  |  |  |  |
|  | Middle frontal gyrus |  | 73 (2) | L |  |  |  |  |  |

|  | **Keywords:** **L/R**: Left or right hemisphere; **MNI**: Montreal Neurological Institute coordinates. Results are significant at p < 0.05 FWE & FDR cluster-corrected in a combination with a threshold of p < 0.001 at the uncorrected voxel level. Only brain regions with >1% cluster overlap were presented. |
| --- | --- |
