# Supplementary figures and images for: Compensation versus deterioration across functional networks in amnestic mild cognitive impairment subtypes
Source: GeroScience. 2024 Oct 5;47(2):1805–22. doi: 10.1007/s11357-024-01369-9 (PMC11978594; doi:10.1007/s11357-024-01369-9)

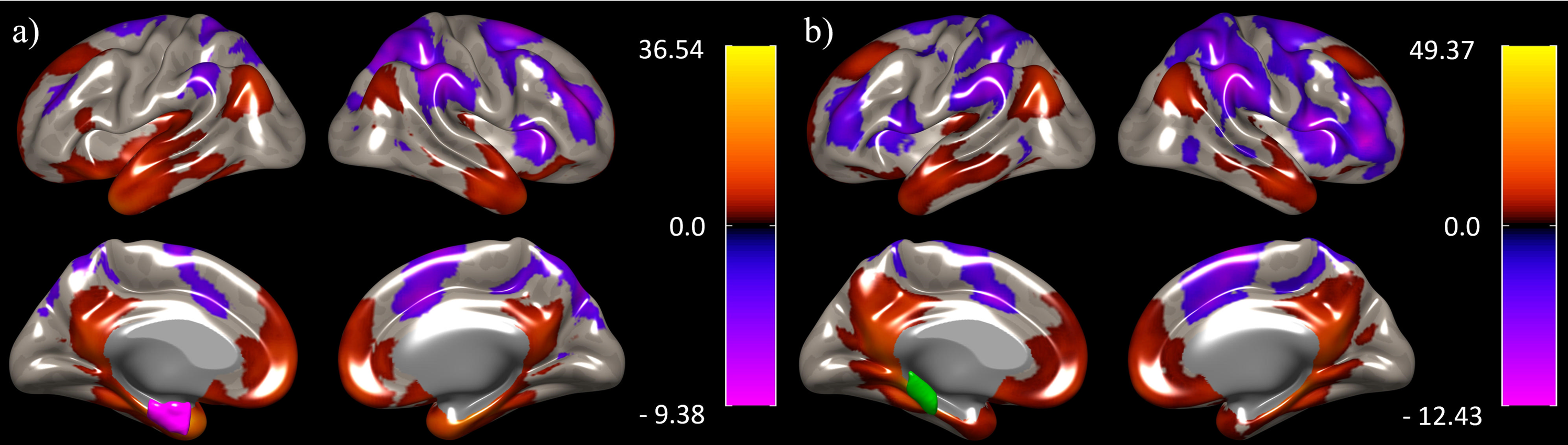

Supplement: Supplementary file 3 — Supplementary file3 (PNG 1.56 MB) [file 11357_2024_1369_Fig4_ESM.png]

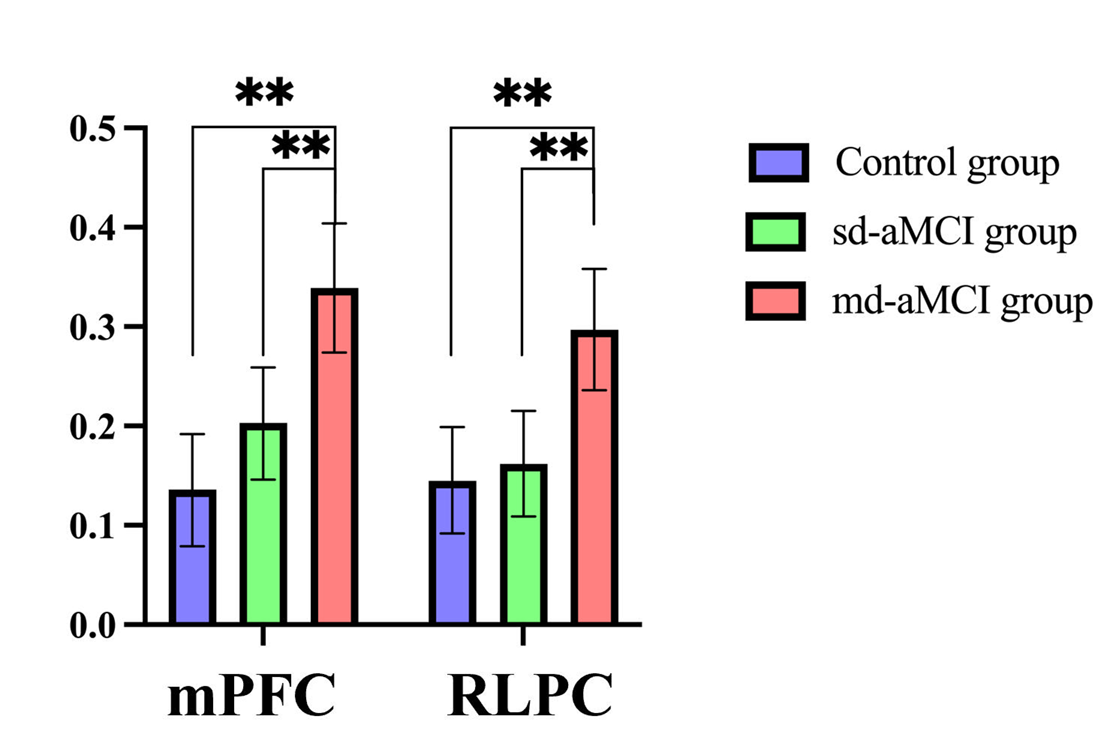

Supplement: Supplementary file 8 — Supplementary file3 (PNG 89.9 KB) [file 11357_2024_1369_Fig5_ESM.png]
