# Supplementary material for: Compensation versus deterioration across functional networks in amnestic mild cognitive impairment subtypes
Source: GeroScience. 2024 Oct 5;47(2):1805–22. doi: 10.1007/s11357-024-01369-9 (PMC11978594; doi:10.1007/s11357-024-01369-9)
Supplement: Supplementary file 5 — Supplementary file4 (DOCX 17 KB) [file 11357_2024_1369_MOESM4_ESM.docx]

| **Table 2.** Brain regions that showed significant functional connectivity differences in the SBA across-group analyses of the left aPHG. | | | | | | | | | | |  |
| --- | --- | --- | --- | --- | --- | --- | --- | --- | --- | --- | --- |
|  | | **Brain region** | **Cluster size** | **# voxels in specific region**  **(% overlap)** | **L/R** | **MNI Coordinates (x,y,z)** | | | **Statistic** | |  |
|  | |  |  |  |  |  | | | **F** | |  |
| **Left aPHG** | | **Group effect** |  |  |  |  |  |  |  |  |  |
|  | | Gyrus rectus | 249 | 136 (18) | R | 8 | 34 | -18 | 12.48 | |  |
|  |  | Superior frontal gyrus (medial orbital part) |  | 41 (5) | R |  |  |  |  |  |  |
|  |  | Medial orbital gyrus |  | 26 (4) | R |  |  |  |  |  |  |
|  |  | Olfactory cortex |  | 13 (5) | R |  |  |  |  |  |  |
|  |  | Anterior orbital gyrus |  | 10 (2) | R |  |  |  |  |  |  |
|  |  | Anterior cingulate cortex (subgenual part) |  | 3 (2) | R |  |  |  |  |  |  |
|  |  |  |  |  |  |  |  |  |  |  |  |
|  | |  |  |  |  |  |  |  |  |  |  |
|  | | **Keywords:** **L/R**: Left or right hemisphere; **MNI**: Montreal Neurological Institute coordinates. Results are significant at p < 0.05 FWE & FDR cluster-corrected in a combination with a threshold of p < 0.001 at the uncorrected voxel level. Only brain regions with >1% cluster overlap were presented. | | | | | | | | | |
