# Supplementary material for: Compensation versus deterioration across functional networks in amnestic mild cognitive impairment subtypes
Source: GeroScience. 2024 Oct 5;47(2):1805–22. doi: 10.1007/s11357-024-01369-9 (PMC11978594; doi:10.1007/s11357-024-01369-9)
Supplement: Supplementary file 6 — Supplementary file6 (DOCX 42 KB) [file 11357_2024_1369_MOESM5_ESM.docx]

| **Table 3.** Brain regions that showed significant functional connectivity differences in the SBA across-group analyses of the left pPHG. | | | | | | | | | | | | | | | | | | |
| --- | --- | --- | --- | --- | --- | --- | --- | --- | --- | --- | --- | --- | --- | --- | --- | --- | --- | --- |
|  | | | | **Brain region** | | **Cluster size** | | **# voxels in specific region**  **(% overlap)** | | **L/R** | | **MNI Coordinates (x,y,z)** | | | | **Statistic** | | |
|  | | | |  | |  | |  | |  | |  | | | | **F** | | |
| **Left pPHG** | | **Group effect** | |  | |  | |  | |  | |  |  |  | | |  |  |
|  | | Superior frontal gyrus (medial part) | | 1066 | | 216 (10) | | R | | 8 | | 50 | 8 | 15.30 | | |  |  |
|  | | Superior frontal gyrus (medial orbital part) | |  | | 152 (18) | | R | |  | |  |  |  | |  |  |  |
|  | | Superior frontal gyrus (medial part) | |  | | 143 (5) | | L | |  | |  |  |  | |  |  |  |
|  | | Superior frontal gyrus (medial orbital part) | |  | | 126 (18) | | L | |  | |  |  |  | |  |  |  |
|  | | Anterior cingulate cortex (pregenual part) | |  | | 120 (19) | | L | |  | |  |  |  | |  |  |  |
|  | | Gyrus rectus | |  | | 95 (11) | | L | |  | |  |  |  | |  |  |  |
|  | | Gyrus rectus | |  | | 93 (12) | | R | |  | |  |  |  | |  |  |  |
|  | | Anterior cingulate cortex (pregenual part) | |  | | 80 (12) | | R | |  | |  |  |  | |  |  |  |
|  | |  | |  | |  | |  | |  | |  |  |  | |  |  |  |
|  | | Superior frontal gyrus (medial part) | | 242 | | 97 (3) | | L | | 4 | | 54 | 38 | 12.12 | | |  |  |
|  | | Superior frontal gyrus (medial part) | |  | | 63 (3) | | R | |  | |  |  |  | |  |  |  |
|  | |  | |  | |  | |  | |  | |  |  |  | |  |  |  |
|  | | Supramarginal gyrus | | 194 | | 141 (11) | | L | | -52 | | -28 | 22 | 13.61 | | |  |  |
|  | |  | |  | |  | |  | |  | |  |  |  | |  |  |  |
|  | **Keywords:** **L/R**: Left or right hemisphere; **MNI**: Montreal Neurological Institute coordinates. Results are significant at p < 0.05 FWE & FDR cluster-corrected in a combination with a threshold of p < 0.001 at the uncorrected voxel level. Only brain regions with >1% cluster overlap were presented. | | | | | | | | | | | | | | | |  |  |
