# Supplementary material for: Compensation versus deterioration across functional networks in amnestic mild cognitive impairment subtypes
Source: GeroScience. 2024 Oct 5;47(2):1805–22. doi: 10.1007/s11357-024-01369-9 (PMC11978594; doi:10.1007/s11357-024-01369-9)
Supplement: Supplementary file 7 — Supplementary file7 (DOCX 15 KB) [file 11357_2024_1369_MOESM6_ESM.docx]

**Table 4.** Mean values and standard errors (SE, in brackets) of the ROI-to-ROI analysis of the left pPHG.

|  |  | | | | | |
| --- | --- | --- | --- | --- | --- | --- |
|  | | **Control group**  ***N* = 30** | **sd-aMCI**  ***N* = 29** | **md-aMCI**  ***N* = 26** | ***p*** | **Post hoc comparison *** |
| **Z-scores** | |  |  |  |  |  |
| Left pPHG-mPFC | | 0.136 (0.029) | 0.203 (0.028) | 0.339 (0.033) | < 0.001 | < .001^b^/.009^c^ |
| Left pPHG-RLPC | | 0.145 (0.027) | 0.162 (0.027) | 0.297 (0.031) | 0.007 | .002^b^/.006^c^ |
| **Abbreviations:** sd-aMCI: Single-domain amnestic mild cognitive impairment, md-aMCI: multiple-domain amnestic mild cognitive impairment, pPHG: posterior parahippocampal gyrus, mPFC: medial prefrontal cortex, RLPC: right lateral parietal cortex. Post-hoc comparisons: ***a*:** Control group *vs* sd-aMCI; ***b***: Control group *vs* md-aMCI group; ***c***: sd-aMCI group *vs* md-aMCI group. *Bonferroni correction. | | | | | | |
