# Supplementary material for: Compensation versus deterioration across functional networks in amnestic mild cognitive impairment subtypes
Source: GeroScience. 2024 Oct 5;47(2):1805–22. doi: 10.1007/s11357-024-01369-9 (PMC11978594; doi:10.1007/s11357-024-01369-9)
Supplement: Supplementary file 10 — Supplementary file8 (DOCX 17 KB) [file 11357_2024_1369_MOESM8_ESM.docx]

**Table 5.** Mean values and standard errors (SE, in brackets) of post-hoc intra/inter-network connectivity analyses.

|  |  | | | | | |
| --- | --- | --- | --- | --- | --- | --- |
|  | | **Control group**  ***N* = 30** | **sd-aMCI**  ***N* = 29** | **md-aMCI**  ***N* = 26** | ***p*** | **Post hoc comparison *** |
| DMN | | 0.304 (0.021) | 0.335 (0.020) | 0.393 (0.023) | 0.043 | 0.019^b^ |
| SAL | | 0.465 (0.032) | 0.500 (0.032) | 0.512 (0.035) | 0.798 | NS |
| DMN-SAL | | - 0.155 (0.21) | - 0.180 (0.021) | - 0.251 (0.023) | 0.009 | 0.013 ^b^ |
| **Abbreviations:** sd-aMCI: Single-domain amnestic mild cognitive impairment, md-aMCI: multiple-domain amnestic mild cognitive impairment. Post-hoc comparisons: ***a*:** Control group *vs* sd-aMCI; ***b***: Control group *vs* md-aMCI group; ***c***: sd-aMCI group *vs* md-aMCI group. *Bonferroni correction. | | | | | | |
